# Supplementary material for: Echinocandin tolerance and persistence in vitro are regulated by calcineurin signaling in Candida glabrata
Source: mBio. 2025 Dec 5;17(1):e02546-25. doi: 10.1128/mbio.02546-25 (PMC12802179; doi:10.1128/mbio.02546-25)
Supplement: Supplemental Figures — Figures S0 to S9. [file mbio.02546-25-s0001.pdf]

Figure S0 Harrington et al

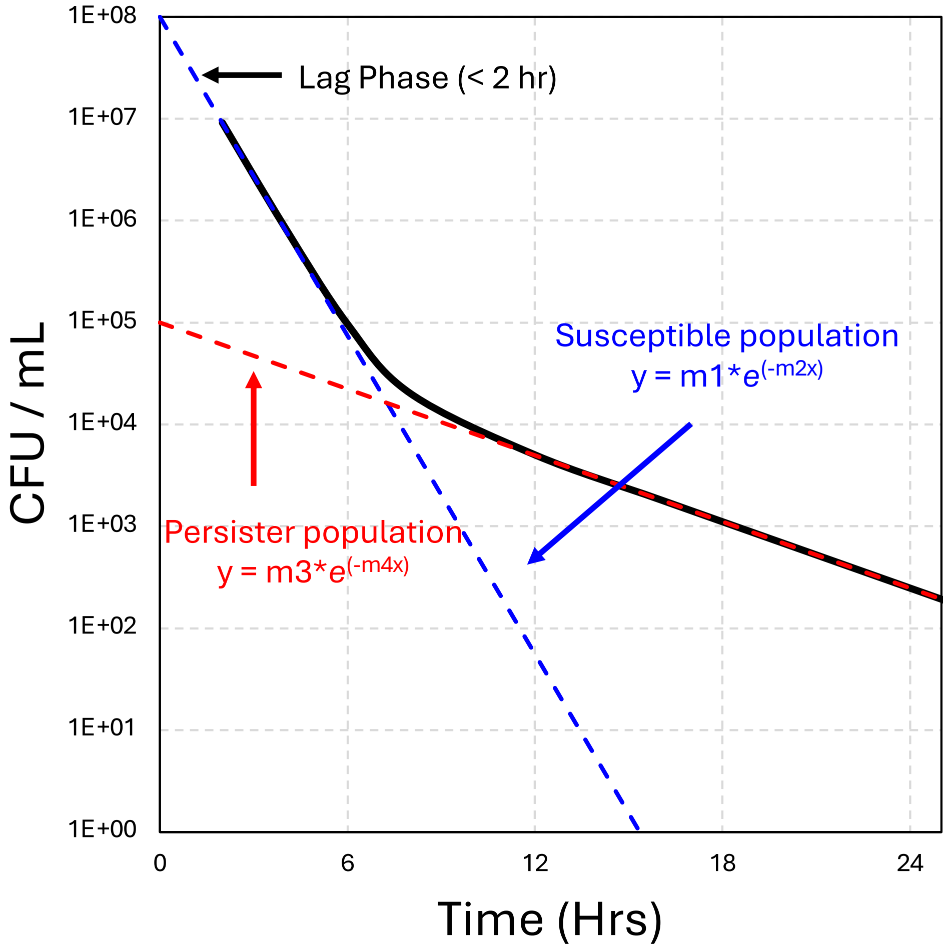


**Fig S0. Analysis of time-kill data.** Four to eight replicate colonies are exposed to supra-MIC doses of antifungals in SCD medium at 30°C. Aliquots are removed periodically, serially diluted, and plated on drug-free SCD medium. Single colonies were counted at one dilution to determine viability (CFU per mL). Replicates were averaged, log-transformed, and fit to the equation y = log(m1$*\, e^{(-\mathrm{m2x})}$+m3 $*\, e^{(-\mathrm{m4x})}$) after removing data points in the lag-phase. Four parameters are estimated: the number of Susceptible and Persister cells at time = 0 (m1, m3) and the kill coefficients (m2, m4) which are converted to half-lives (= ln2/(m2 or m4)). The smooth curve (solid black) represents a typical cure fit that is the sum of the Susceptible (dashed blue) and Persister (dashed red) components.

Figure S1     Harrington et al

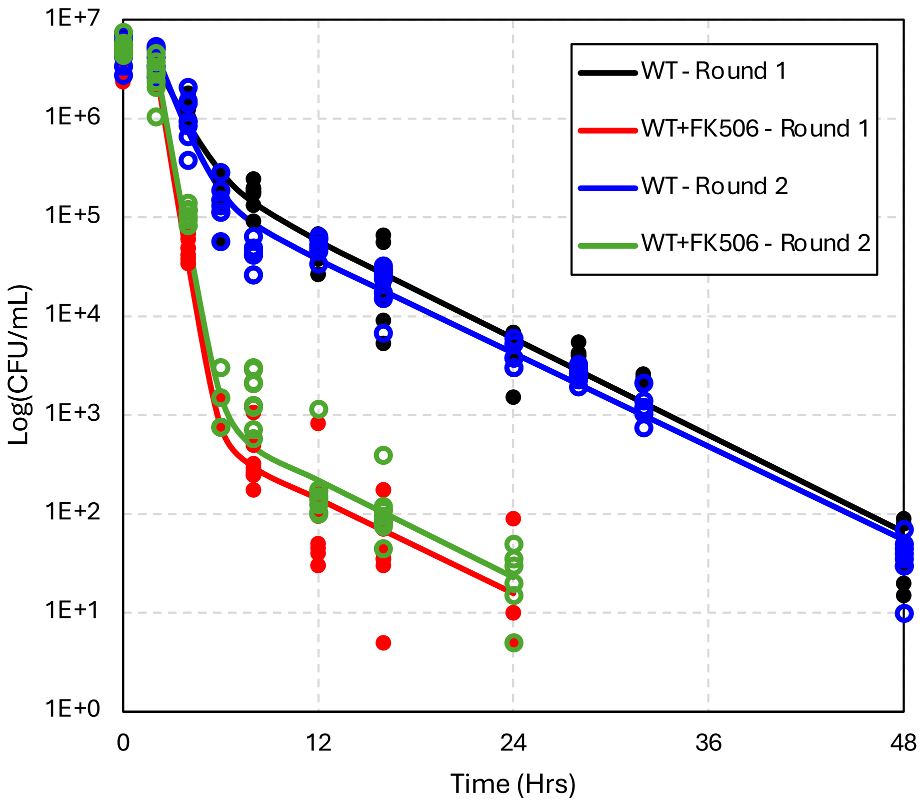


**Fig S1. Biological replicates experience similar responses to micafungin and FK506 treatment.** Eight single colonies of wild-type BG14 cells were grown to saturation for 72 hours, then diluted 50-fold into media containing micafungin (0.125 µg/mL) medium containing or lacking FK506 (1 µg/mL) and sampled as described in Fig. 1. Here, individual replicate cultures were counted and plotted (symbols) were plotted with curve fits imported from Fig 1.

Figure S2        Harrington et al

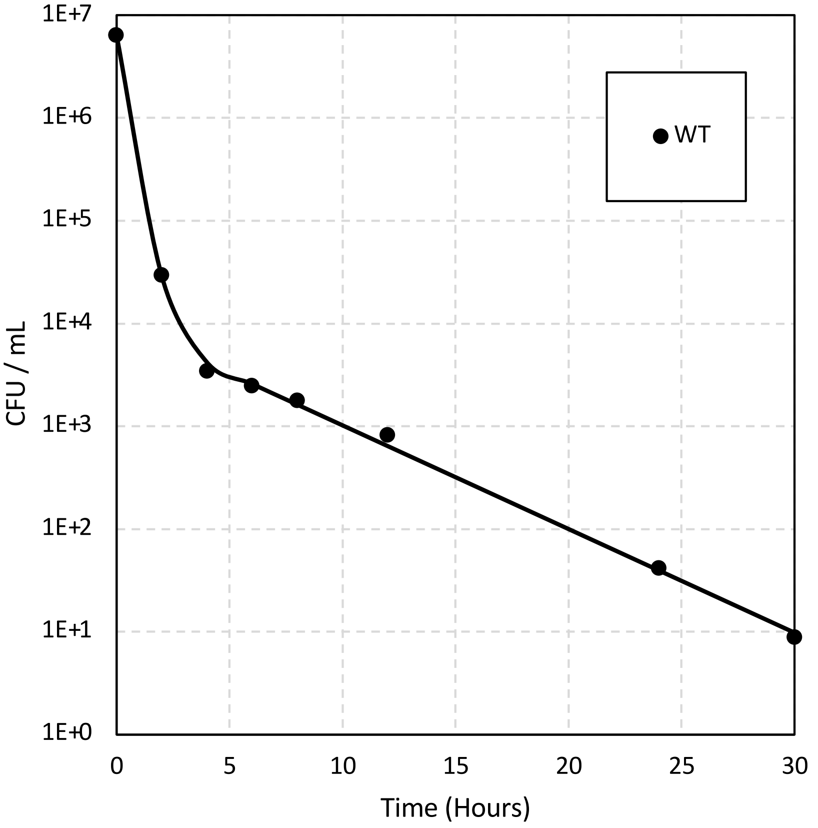


**Fig S2. Log-phase populations exhibit no lag phase in time-kill experiments.** Four single colonies of wild-type strain BG14 were grown in fresh SCD medium for 24 hours until log phase phase was achieved. Time-kill assays with micafungin were performed as described in Fig. 1.

Figure S3 Harrington et al

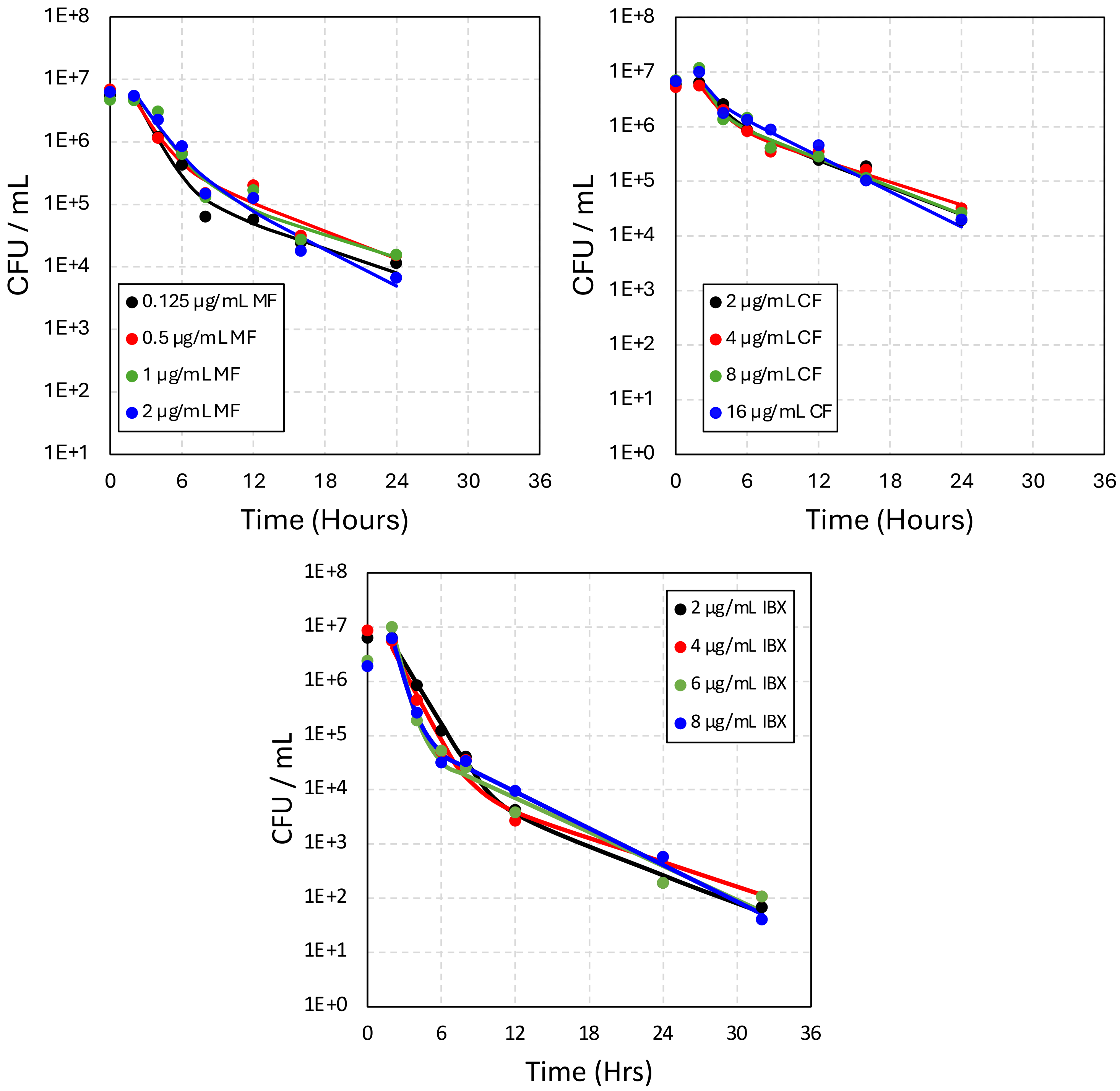


**Figure S3. Tolerance and persistence to β-1,3-glucan synthase inhibitors is dose- and drug-independent.** Wild-type BG14 cells were grown and treated with varying concentrations of micafungin (MF), caspofungin (CF), or ibrexafungerp (IBX) in as described Fig 1. Data points are the averages of four biological replicates and were used to generate the curve fits.

Figure S4 Harrington et al

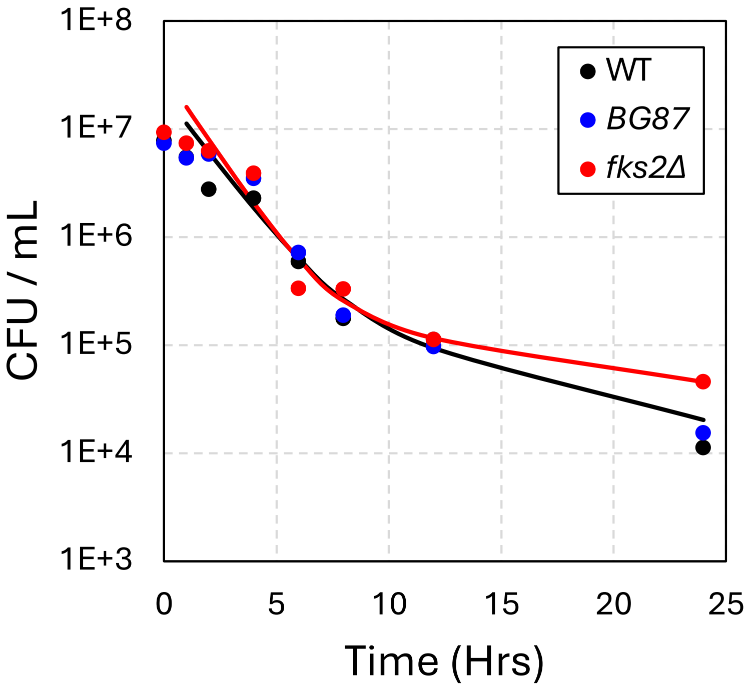


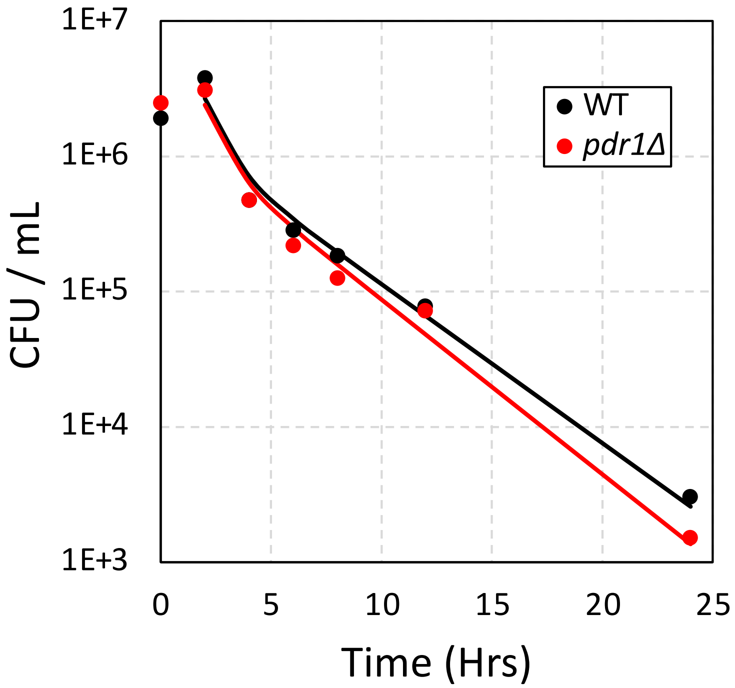


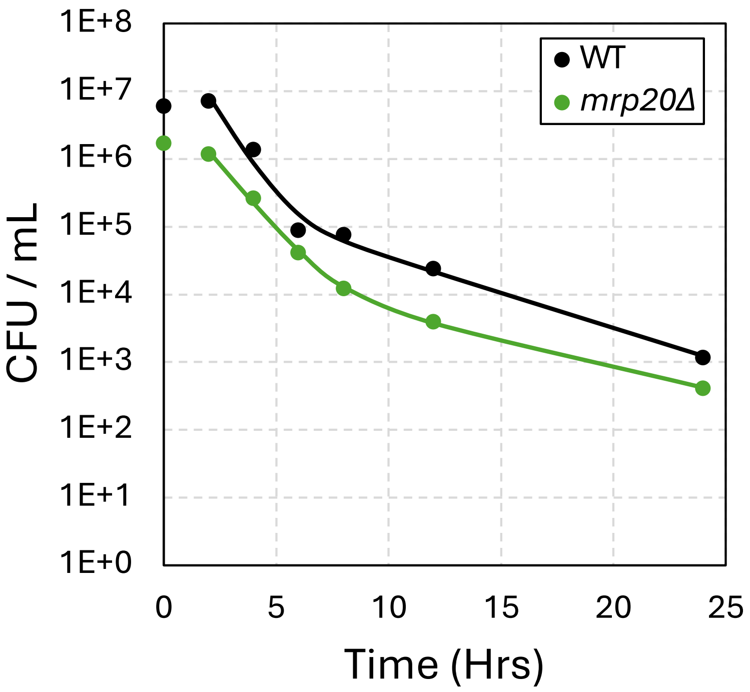


**Figure S4. Strains associated with increased and decreased micafungin resistance do not alter tolerance or persistence.** (Top Row) Wild-type strain BG14 (black) and the derived strains (colored symbols) were grown and treated with micafungin as described Fig 1. (Bottom) *mrp20Δ* mutant was treated with an 8X dose (1 μg/mL) of micafungin to compensate for resistance. Cultures were sampled and CFUs were calculated as described in Fig. 1. Data points are the averages of four biological replicates and were used to generate the curve fits.

Figure S5        Harrington et al

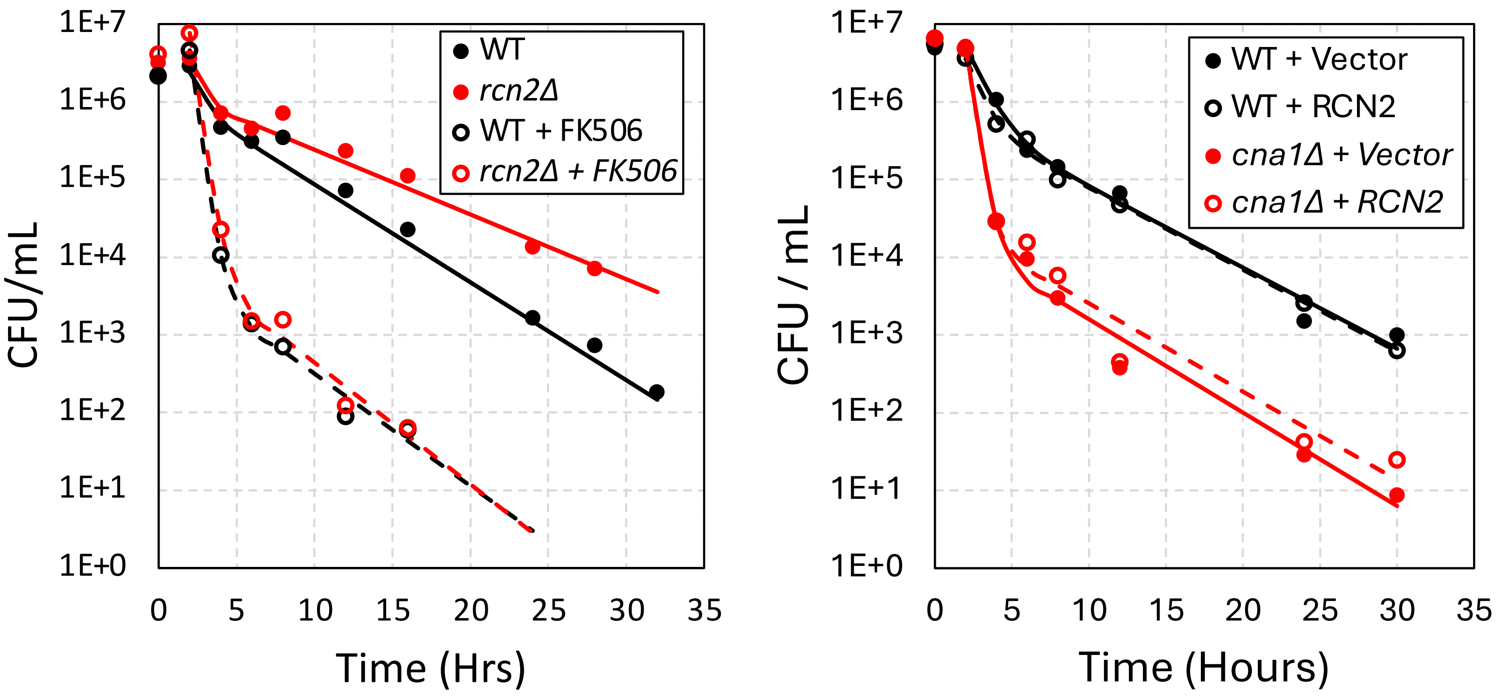


**Figure S5. *RCN2* negatively regulates calcineurin in time-kill experiments.** (Left) Wild-type strain BG14 (black) and derived *rcn2Δ* mutant (red) were grown and treated with micafungin containing or lacking FK506 and time-kill experiments were performed as described Fig 1. Data points are the averages of four biological replicates and were used to generate the curve fits. (Right) Wild-type strain BG14 (black) and *cna1Δ* mutants (red) were transformed with either empty plasmid (+ vector) or pCN-PDC1-RCN2 (+ *RCN2*). Single colonies were grown to saturation in SCD+NAT media for 72 hours to select for plasmid, then washed and diluted into fresh medium containing micafungin and and analyzed as described in Fig. 1 . Data points are the averages of four biological replicates and were used to generate the curve fits.

Figure S6        Harrington et al

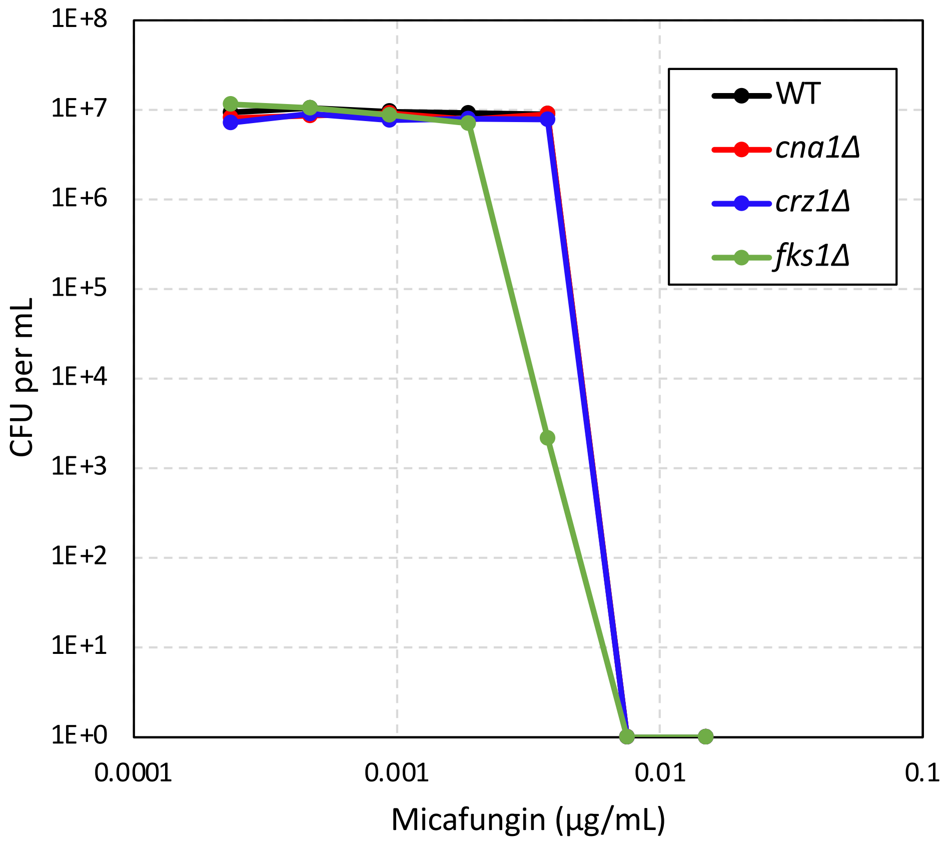


**Figure S6. Heteroresistance to micafungin was not observed in *C. glabrata* strains.** Wild-type strain BG14 (black) and mutant strains (colors) were grown to saturation for 72 hours, diluted into fresh SCD medium, and then plated on YPD agar media containing varying doses of micafungin (0 to 0.015 µg/mL). Plates were incubated for 24 hours at 30°C and colonies were counted manually using a dissecting microscope.

Figure S7 Harrington et al

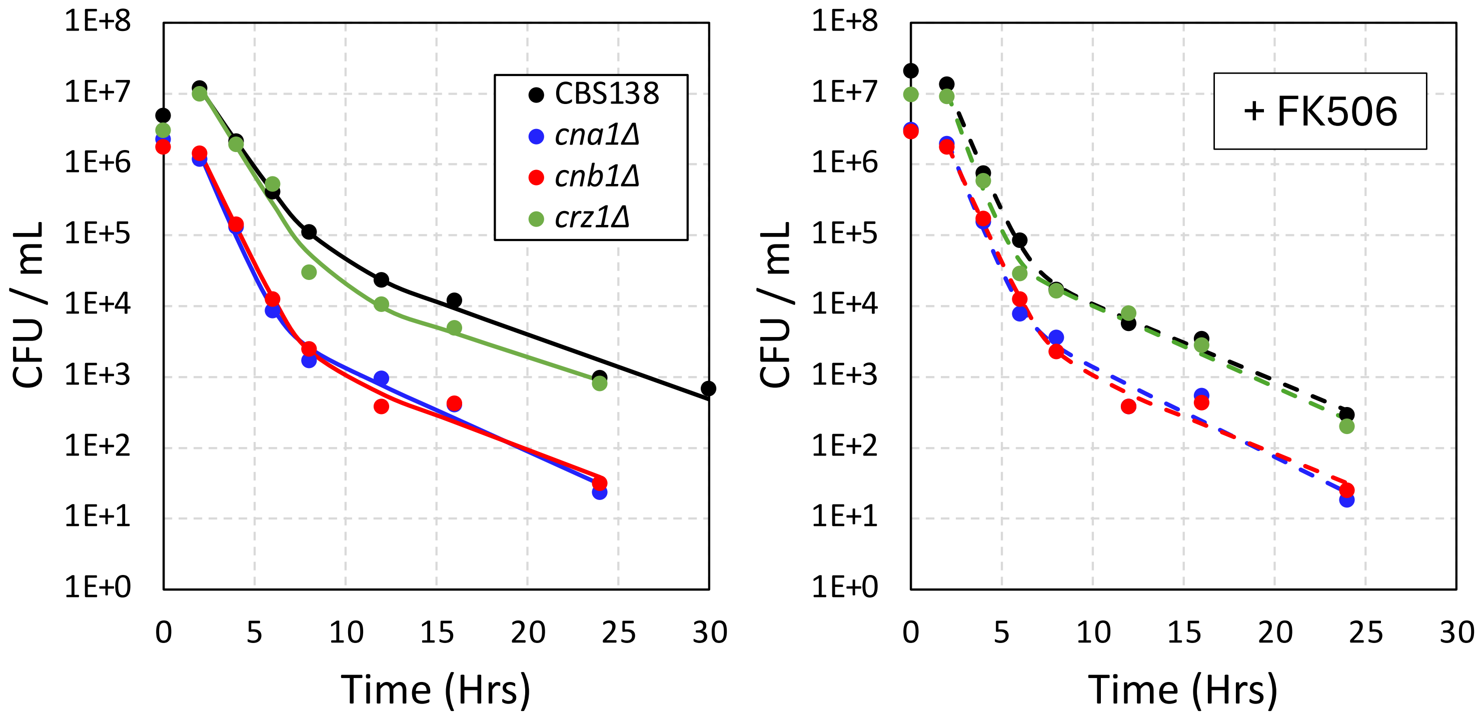


**Figure S7. CN-dependent tolerance and persistence is conserved in the CBS138 strain background of *C. glabrata*.** Wild-type strain CBS138 (black) and derived mutant strains (colors) were grown and treated with micafungin containing or lacking FK506 as described Fig 1. Data points are the averages of four biological replicates and were used to generate the curve fits.

Figure S8        Harrington et al

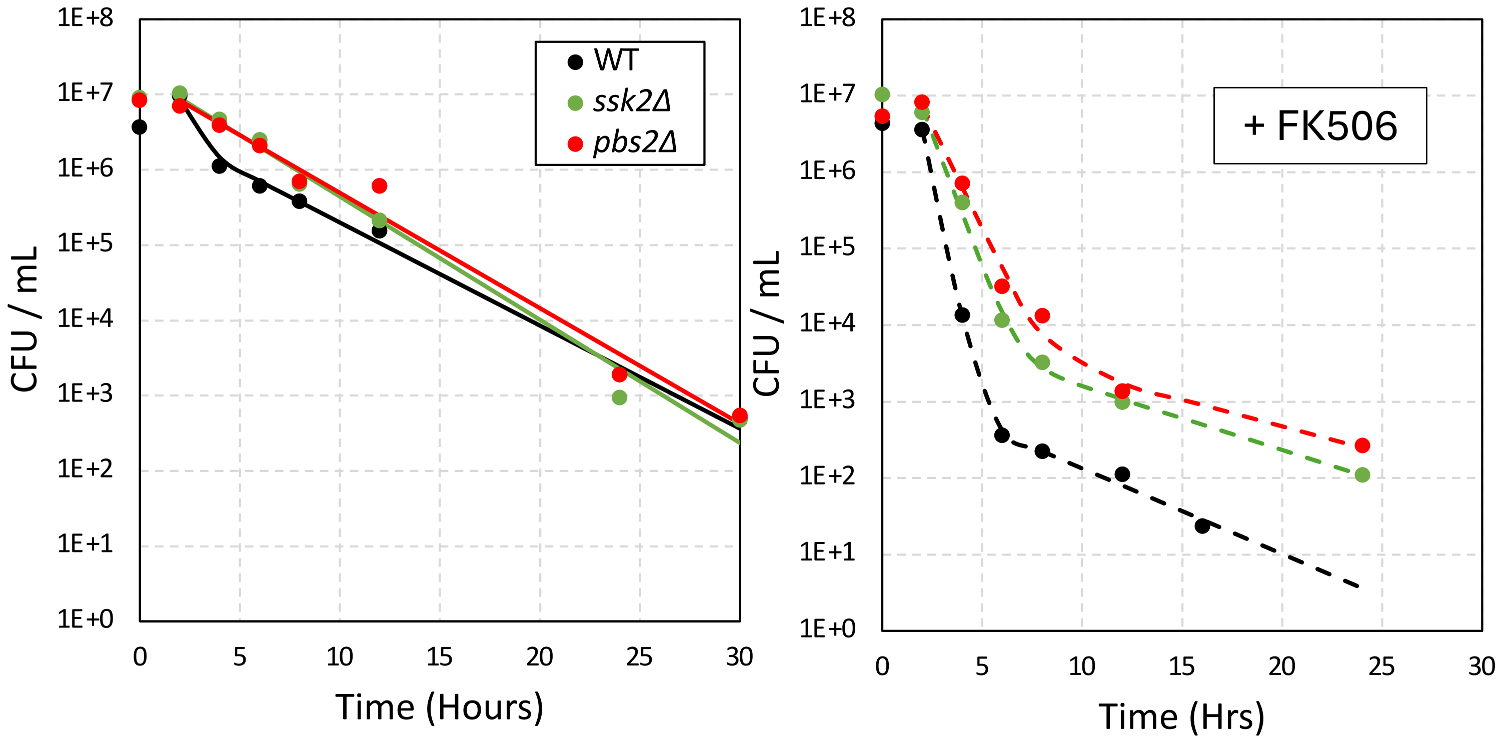


**Figure S8. The HOG pathway negatively regulates tolerance and persistence independent of calcineurin in BG2 strains.** Wild-type strain BG2 (black) and derived mutant strains (colors) were grown as describe in Fig. 1, treated with a higher dose of micafungin (1 µg/mL) containing or lacking FK506 (1 µg/mL) , and sampled as previously described. Data points are the averages of four biological replicates and were used to generate the curve fits.

Figure S9 Harrington et al

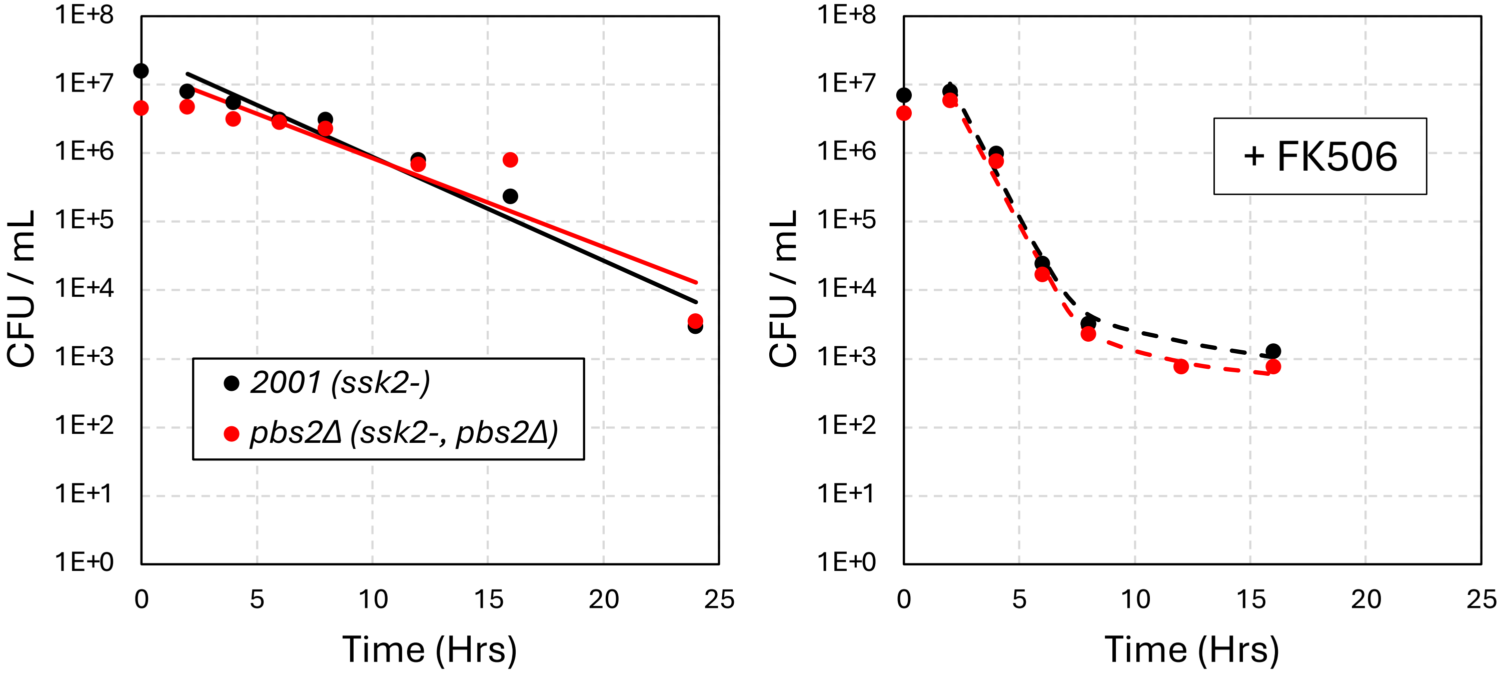


**Figure S9. CBS138-derived strains naturally lack HOG signaling.** Wild-type strain 2001 (derived from CBS138 with natural *ssk2*- mutation; black) and a *pbs2Δ* mutant derivative (red) were grown, treated with a high dose of micafungin (1 μg/mL) containing or lacking FK506 (1 μg/mL), and sampled as described in Fig. 1. Data points are the averages of four biological replicates and were used to generate the curve fits.
